# The Gut Microbiota and Irritable Bowel Syndrome: Friend or Foe?

**DOI:** 10.1155/2012/151085

**Published:** 2012-04-22

**Authors:** Uday C. Ghoshal, Ratnakar Shukla, Ujjala Ghoshal, Kok-Ann Gwee, Siew C. Ng, Eamonn M. M. Quigley

**Affiliations:** ^1^Department of Gastroenterology, Sanjay Gandhi Postgraduate Institute of Medical Science, Lucknow 226014, India; ^2^Department of Microbiology, Sanjay Gandhi Postgraduate Institute of Medical Science, Lucknow 226014, India; ^3^Stomach, Liver and Bowel Clinic, Gleneagles Hospital, Singapore; ^4^Department of Medicine and Therapeutics, Institute of Digestive Disease, Prince of Wales Hospital, Chinese University of Hong Kong, Hong Kong; ^5^Department of Medicine, Alimentary Pharmabiotic Centre, University College Cork, Cork, Ireland

## Abstract

Progress in the understanding of the pathophysiology of irritable bowel syndrome (IBS), once thought to be a purely psychosomatic disease, has advanced considerably and low-grade inflammation and changes in the gut microbiota now feature as potentially important. The human gut harbours a huge microbial ecosystem, which is equipped to perform a variety of functions such as digestion of food, metabolism of drugs, detoxification of toxic compounds, production of essential vitamins, prevention of attachment of pathogenic bacteria to the gut wall, and maintenance of homeostasis in the gastrointestinal tract. A subset of patients with IBS may have a quantitative increase in bacteria in the small bowel (small intestinal bacterial overgrowth). Qualitative changes in gut microbiota have also been associated with IBS. Targeting the gut microbiota using probiotics and antibiotics has emerged as a potentially effective approach to the treatment of this, hitherto enigmatic, functional bowel disorder. The gut microbiota in health, quantitative and qualitative microbiota changes, and therapeutic manipulations targeting the microbiota in patients with IBS are reviewed in this paper.

## 1. Introduction

Functional bowel disorders, including irritable bowel syndrome (IBS), are common gastrointestinal disorders all over the world. Previously, IBS was thought to be a psychosomatic disorder. However, in the last few decades, advances in our understanding of the pathophysiology of IBS have revealed several factors, including alterations in the microbiota, as potentially relevant to the cause of this syndrome and the precipitation of its symptoms. Indeed, alterations in the gut microbiota are being increasingly implicated in the pathogenesis of several gastrointestinal and systemic diseases. We wish, therefore, to review the gut microbiota and its alterations in, and relationships to, IBS.

## 2. Gut Microbiota in Health

The human gut harbours a huge microbial ecosystem, which is equipped to perform a variety of functions such as the digestion of food, metabolism of the drugs, detoxification of toxic compounds, production of essential vitamins, prevention of attachment of pathogenic bacteria to the gut wall and maintenance of homeostasis in the gastrointestinal tract (GIT) [1–3]. The human gut is first colonized at birth; this microbiota gradually increases in size and diversity up to the end of the first year of life; by that time, the gut microbiota has come to resemble that of the adult and remains relatively stable thereafter [4]. The composition of the gut microbiota varies according to age, sex, diet, geographical origin of the individual and is also influenced by certain environmental factors, such as administration of antibiotics [1]. The composition and activity of the human gut microbiota affect gastrointestinal and systemic homeostasis. There are 10 times more microbial cells (10^14^) in the gut than cells in the entire body (10^13^) [3]. A recent study has suggested that the human gut microbiota consists of more than 35,000 bacterial species and that 70% of all of microbes in the human body reside in the colon [4]. The small intestine consists of mainly Gram-positive and aerobic bacteria, whereas the large intestine consists largely of Gram-negative and strictly anaerobic bacteria [5, 6].

## 3. Alteration of Gut Microbiota

Alterations in the normal gut microbiota have been suggested as etiologic factors in the development of functional gastrointestinal disorders such as IBS and functional dyspepsia, common GI disorders of unknown etiology [7–9]. Quantitative alterations in the gut microbiota in the small bowel may result in the clinical syndrome recognized as small intestinal bacterial overgrowth (SIBO) [10]. In SIBO, such is the change in the number and type of bacteria in the upper small intestine that diarrhea, abdominal bloating, malabsorption, abdominal pain, and excessive gas production result; severe motor dysfunction may be an underlying cause [11–13].

Quantitative changes in the colonic microbiota may lead to the proliferation and development of specific species that produce more short-chain fatty acids (SCFAs) and gases, such as methane, hydrogen, and carbon dioxide, potentially resulting in abdominal bloating and distension. An increase in the concentration of SCFAs (acetate, butyrate, and propionate) leads to acidification of the colon and deconjugation of bile acid. This in turn may cause significant changes in water and electrolyte transport in the colon which result in diarrhea [8, 14, 15]. Malabsorption of carbohydrates may cause increased production of hydrogen gas, which is associated mainly with diarrhea-predominant IBS (IBS-D) [16] whilst excess methane gas production is associated with constipation-predominant IBS (IBS-C) [14].

## 4. Irritable Bowel Syndrome

IBS is a functional gastrointestinal disorder associated with abdominal discomfort or pain, distension and bloating, diarrhea, constipation, or mixed bowel habits (i.e., both constipation and diarrhea; IBS-M). IBS subjects may also experience greater levels of stress, anxiety, and depression compared to healthy subjects [16, 17]. All of these co-morbidities are associated with impaired quality of life in IBS patients [18–20]. Several diagnostic criteria (Kruis, Manning, Rome) have been used to distinguish IBS patients from those with organic bowel disease in daily clinical practice [11, 21].

The prevalence of IBS varies from 9% to 22% in the United States and Europe [22, 23]. In Asian countries, IBS affects 4% to 20% of populations. In Asia, the lowest prevalence has been reported from India, at 4.2%, and the highest from Japan and Singapore [23–25].

The exact etiology and pathophysiology of IBS remain unclear. Several hypotheses have been proposed which include alteration in the gut microbiota, dysregulation of the brain-gut axis and autonomic nervous system, visceral hypersensitivity, and altered levels of gastrointestinal neuropeptides and hormones [11, 22, 26]. Furthermore, abnormal gastrointestinal motility, as well as genetic, environmental, and psychological factors, may also play important roles in the development of IBS. Recent studies have also shown that IBS is associated with low-grade intestinal inflammation resulting from an activated immune system, in response to a normal or abnormal gut microbiota [1, 27, 28].

## 5. Pathophysiology

### 5.1. Altered Gut Microbiota or Dysbiosis

There is a growing interest in investigating the role of an altered gut microbiota in the pathogenesis of IBS [14, 29]. Normal gut microbiota have either direct bactericidal effects or can prevent the adherence of pathogenic bacteria to the wall of the gastrointestinal tract [7, 30, 31]. Dysbiosis in the gut may facilitate the adhesion of enteric pathogens in the human gut which can be associated with IBS symptoms [30, 32]. Alteration in the composition of the normal microbiota and disturbed colonic fermentation in IBS patients may play an important role in development of IBS symptoms [33]. Firmicutes and Bacteroidetes are the major beneficial gut microbiota in the normal human and changes in their relative numbers have been reported in IBS patients [1]. Significantly, a two-fold increase in the ratio of Firmicutes to Bacteroidetes has been reported in IBS patients. This results from an increase in the quantity of *Ruminococcus, Clostridium, Dorea* species and a decrease in the quantity of *Bifidobacterium and Faecalibacterium* species [34]. Significantly greater abundance of classes of Gammaproteobacteria and Enterobacteriaceae in healthy controls were positively correlated with the inflammatory markers IL-6 and IL-8. Higher levels of these cytokines have been reported in IBS patients [34, 35]. Due to a different microenvironment in the intestinal epithelium and lumen, the composition of microbiota is not the same at the level of the gut epithelium and lumen [33]. Culture-based analysis had shown that there were significant differences between the mucosa-associated microbiota and fecal samples in both healthy control and IBS patients. For example, a significantly increased quantity of aerobic bacteria and *Lactobacillus* was noted in IBS-D in feces but not in mucosal samples [19].

Several studies using different methodologies to define changes in the microbiota have been published, including classic culture-based techniques, PCR-based molecular technologies such as real-time PCR (q PCR), microarray, DGGE (denaturation gradient gel electrophoresis), GC profiling, and high-throughput sequencing based on 16 s rRNA [33, 36] (see Table 1). 

Bacterial fermentation of undigested, unabsorbed food causes production of SCFAs, which are bacteriostatic for a group of species, either directly or by reducing pH [2, 7, 30, 31, 37]. SIBO causes unusual fermentation with increases in gas production and abnormal gastrointestinal motility. A previous study reported that the prevalence of SIBO in IBS subjects detected by lactulose hydrogen breath test was about 78% [14]. However, this study used the lactulose hydrogen breath test which has now been shown to be prone to a high rate of false positive result [38] and may have led to an overestimation of its frequency.

### 5.2. Postinfectious IBS (PI-IBS)

IBS develops in a subgroup of individuals following an episode of acute gastrointestinal infection, known as PI-IBS. Acute enteric infections are characterized by abdominal discomfort, fever, vomiting, bloating, and diarrhea, [14, 39, 40]. Fever and vomiting generally improve after a few days while abdominal discomfort, diarrhea and bloating “persist” in those who develop PI-IBS [39]. Risk factors for the development of PI-IBS include younger age, female gender, prolonged duration of diarrhea, the presence of bacterial pathogens, and psychological morbidities including anxiety, frustration, and depression [41–43].

The risk of IBS increased sixfold after an acute gastrointestinal infection and persists for up to 3 years [44, 45]. Recent studies have shown that PI-IBS develops in 3–30% of individuals with bacterial gastroenteritis caused by a number of enteric pathogens such as *Campylobacter species, Clostridium perfringens, Staphylococcus aureus, Bacillus cereus, Shigella *and viruses [7, 30, 32, 39].

The prevalence of PI-IBS in subjects who had suffered from diarrhea whilst travelling in developing countries has been estimated to be 7–14% [46]. The diagnosis of PI-IBS was made according to Rome criteria for IBS among individuals who did not have IBS previously but developed it after the episode of acute gastroenteritis [14, 39]. Recent studies have suggested that PI-IBS is associated with dysbiosis in the gut (induced either by enteric infection or by the use of antibiotics), psychological factors (such as stress, anxiety, and depression), genetic susceptibility of the host, persistent activation of the host immune system, increased intestinal permeability, and an increased number of enterochromaffin cells in the gut [22, 39, 47].

Potentially harmful microorganisms and their metabolites cause the disruption of tight junctions between epithelial cells leading to increased mucosal permeability during the acute phase of infection [48, 49]. The permeability of the proximal small intestine in PI-IBS was shown to be significantly increased compared to controls [50]. Zona occludens (ZO-1) are tight junction protein that link the transmembrane domain. A lesser expression of ZO-1 has been observed in biopsy samples of IBS subjects [51].

As a result of increased permeability of intestinal epithelial cells, an influx of immune cells including mast cells, T lymphocytes, macrophages, and inflammatory mediators such as IL-2, IL-1*β*, IL-6, IL10, TGF-*β* occurs after an acute enteric infection [48, 49, 52, 53]. A growing body of evidence indicates that mast cells and lymphocytes are increased in the mucosa of both PI-IBS and IBS patients [54]. T lymphocytes are significantly increased in PI-IBS compared with control subjects [52]. IL-1*β* is significantly increased in PI-IBS patients during and after the infection compared with those subjects who do not develop PI-IBS [53]. Certain cytokines such as IL-6 may also further change gut permeability. Increased levels of IL-6 have been reported in IBS subjects compared with healthy controls [49]. These inflammatory mediators (IL-1*α*, IL-1*β*, IL-6, IFN-*γ*) damage the intestinal epithelial barrier and cause inflammation [48]. Furthermore, the administration of antibiotics altered the composition of the gut microbiota and inhibited the expression of antimicrobial peptide Reg III *γ* produced by commensal bacteria. Reg III *γ* strengthens the intestinal epithelial barrier by inhibiting Gram-negative pathogens but has no effect on Gram-positive bacteria; its inhibition results, therefore, in a favourable environment for enteric pathogens to proliferate [4, 7, 55].

### 5.3. Small Intestinal Bacterial Overgrowth (SIBO)

Normally, the density of bacteria is much lower in the small intestine than in the large intestine. There are 10^10–12  ^ colony-forming units (cfu) per mL, 10^5–8^ cfu/mL, 10^0–5^ cfu/mL and 10^0–4^ cfu/mL bacteria in the cecum, terminal ileum, proximal ileum, and jejunum/duodenum, respectively [54, 56]. The major families of bacteria in the small intestine include Bacilli, Streptococcaceae, Actinobacteria, Actinomycinaea, and Corynebacteriaceae [4]. Qualitative or quantitative changes in the microbiota of small intestine may lead to the clinical features of SIBO [56, 57]. In healthy individuals, the normal bacterial count in the proximal small intestine is ≤10^4^ cfu/mL; SIBO is traditionally defined by a bacterial count of ≥10^5^ cfu per mL in jejunal aspirate [2, 56]. Several techniques have been used for the diagnosis of SIBO, which include the lactulose hydrogen (LHBT), ^14^C xylose and glucose hydrogen (GHBT) breath tests and culture of jejunal aspirate [13, 56, 58]. Hydrogen and methane are normally produced in the large intestine but in case of SIBO these gases are produced in the small bowel also [13]. *Methanobrevibacter smithii*, *Methanobrevibacter stadtmanae,* and, possibly, *Coliform* bacteria produce methane gas [13, 14].

The jejunal aspirate culture has, traditionally, been used as the gold standard to diagnose SIBO. The limitations of this test however include invasiveness and the challenges posed by attempting to culture all strains and species [54, 57]; therefore, hydrogen breath tests (GHBT or LHBT) are most commonly used [59]. In a study where endoscopic jejunal biopsy culture was used to diagnose SIBO, its sensitivity and specificity were 83.5% and 97.2%, respectively [60]. The prevalence of SIBO in IBS patients was 4% (based on the definition of ≥10^5 ^cfu/mL of bacteria in jejunal aspirate) and no different from that seen in healthy individuals [56]. In contrast, in a study of 111 IBS subjects using LHBT, Pimentel et al. reported a prevalence of SIBO of 84% in IBS (in comparison to 20% in healthy individuals). Furthermore, the administration of neomycin significantly alleviated IBS symptoms [61]. Recent published data have shown that, using jejunal aspirate as the gold standard, double-peak in LHBT only diagnoses one-third of SIBO patients; the sensitivity and specificity of LHBT were 31% and 86% and those of GHBT were 44% and 80%, respectively [62]. In a separate study by Berthold, the sensitivity and specificity of lactose-(13C) ureide breath test (LUBT) were 66.7% and 100% and those of GHBT 41.7% and 44.4%, respectively [63]. The sensitivity and specificity after glucose were 62.5% and 82% and after lactulose were 52% and 86%, respectively [13]. The variation in LHBT and GHBT may be due to differences in the diagnostic criteria used for selection of IBS patients, ecological origin, nature of the substrate used, and diagnostic methods [59].

 The administration of antibiotics and probiotics reduced not only gas-related problems but also IBS-like symptoms [54, 56, 61, 64]. SIBO may arise due to hypochlorhydria, altered intestinal motility [2, 56], altered bacteriostatic properties of pancreatic and biliary secretion [65], and a dysregulated immune response [2]. Proton pump inhibitors (PPIs) may predispose to SIBO by decreasing acid secretion in the stomach [66]. Theisen reported that the inhibition of acid secretion related to the administration of omeprazole led to an increased concentration of unconjugated bile acids. Deconjugation of bile acids inhibits the absorption of fat and lipid soluble vitamins [67]. Thus, the potential side effects of PPI include constipation, diarrhea, bloating, and abdominal pain, symptoms which resemble those of IBS [68] (see Table 2).

### 5.4. Intestinal Barrier Dysfunction and Altered Immune Response

Commensal bacteria provide a favourable environment, prevent the adherence of pathogenic bacteria, and modulate innate and adaptive immune responses [55, 69]. They also protect the intestinal epithelial cell (IEC) from an inflammatory response. The intestinal epithelium contains a large number of lymphocytes that can remove infected epithelial cells [70].

Paneth cells found at the base of the intestinal gland (crypt of Lieberkuhn) throughout the small intestine can prevent the penetration of intestinal epithelium by commensal and pathogenic bacteria [71]. IECs are protected by a glycocalyx layer of mucus, the epithelial junction adhesion complex, secretory IgA, chloride secretion, and other glycoproteins [30, 55]. The intestinal barrier consists of the tight junction complex, adherin junctions, gap junctions, and desmosomes. There are more than 40 types of proteins in the tight junction complex, which play crucial role in maintaining the permeability of the intestinal epithelium [72]. A leaky intestinal epithelium has been reported in subjects with IBS, ulcerative colitis, Crohn's disease, celiac disease, and food-borne infections [72–75]. A recent study has shown that MicroRNA-29a regulates the permeability of the intestine through the generation of glutamine synthetase in patients with IBS. Glutamine synthetase controls the concentration of glutamine. Decreased concentration of glutamine leads to an increase in the permeability of intestinal epithelial cells (IEC), whilst the permeability of IECs can be recovered by the supplementation of glutamine in patients with IBS [75]. The administration of probiotics, fermented milk (*Streptococcus thermophilus, Lactobacillus bulagaris Lactobacillus acidophilus, and Bifidobacterium longum*) and *L. plantarum* has been shown to strengthen the intestinal barrier [72–74]. Paneth cells and enterocytes in the gut secrete antimicrobial molecules such as angiogenin 4, defensins, IgA antibodies, and RegIII *γ*. These antimicrobial peptides destroy pathogenic bacteria by forming a pore in the bacterial cell wall [7, 55, 76]. These data suggest that a symbiotic relationship is present between commensal bacteria and the host.

The composition of the gut microbiota influences the development of the immune system. Any alterations in the gut microbiota due to enteric infections, antibiotic therapy or acid suppressive treatment lead to activation of both the innate and adaptive immune responses [69]. Certain commensal bacteria induce intestinal inflammation while others regulate the immune response. Commensal bacteria from the phylum of Bacteroidetes and Firmicutes have been shown to induce T regulatory cells and inhibit Th17-mediated inflammation. In contrast, the administration of *Bifidobacterium animalis *subspecies inhibited intestinal inflammation via a reduction of the commensal bacteria Enterobacteriace [55, 77, 78].

**Figure 1:**
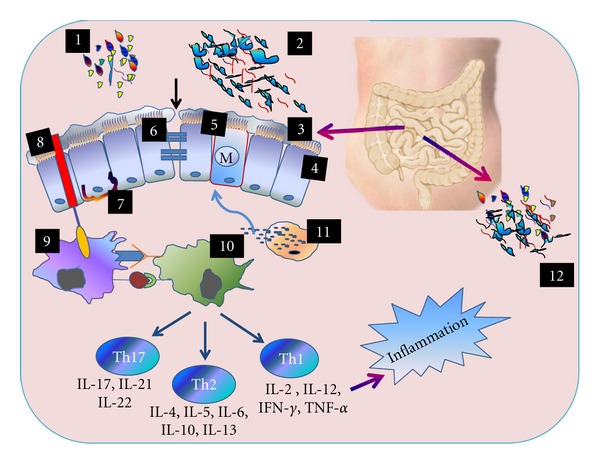
(1) Commensal bacteria (2) Pathogenic bacteria (3) Mucus layer (4) Intestinal epithelium (5) Peyer's patch (6) Tight junction protein (7) Paneth cell (8) Toll-like receptors (9) Dendritic cell (10) T cell (11) Degranulation of mast cells (12) Small intestinal bacterial overgrowth. The intestinal microbes may form a natural barrier to pathogenic bacteria. Therefore, any qualitative or quantitative change in the gut microbiota leads to the instability of the gut microbial ecosystem. It facilitates the entry of pathogenic bacteria and allows them to adhere to the wall of the intestinal epithelial cell. Degranulation of mast cells releases substances that increase the permeability of mucosa resulting in a reduction in the integrity of the tight junctional protein complex. Luminal bacteria or bacterial products such as peptidoglycans and lipopolysaccharides interact with Toll-like receptors on dendritic cells and macrophages. After processing, these cells present the antigen to T cells leading to the production of cytokines, chemokines which cause inflammation in the gastrointestinal tract. Paneth cells are found throughout the small intestine and secrete alpha defensins and lysozyme which, not only eliminate pathogenic bacteria, but also maintain the integrity of the intestinal membrane. Lymphocytes are found in a more organized structure called lymphoid follicles. M cells play an important role in transporting bacteria and microbial particles from the lumen to the lymphoid follicles. The areas around M cells, called Peyer's patches, facilitate the mucosal immune response.

Thus, the gut microbiota plays an important role in the maintenance of homeostasis of various subpopulations of T cells: regulatory T cells (Tregs), T helper 1 (Th1), and T 17 (Th17) cells in the gut [79]. Low-grade inflammation in the intestine in IBS patients is associated with the activation of T lymphocytes and mast cells, increased expression of proinflammatory cytokines such as IL-6 and IL-8, and elevated levels of IL-1*β*, TNF-*α*, and IL-8 in peripheral blood mononuclear cells [80, 81]. Significantly increased levels of TNF-*α*, IL-1*β*, and IL-6, stimulated by lipopolysaccharide (LPS), have been reported in an IBS-D subgroup while increased levels of LPS-stimulated IL-1*β* were described in an IBS-C subgroup (Figure 1).

In PI-IBS, LPS-induced cytokines (TNF-*α*, IL-1*β*, and IL-6) are significantly increased when compared with controls. IL-1*β* causes alteration in secretomotor function during inflammation [80]. Increased concentrations of IL-1*β* are associated with the development of IBS symptoms such as alteration in bowel habits [81]. Elevated levels of IL-6 are produced during stress, inflammation, and infectious disease [82]. Thymus-derived T regulatory cells (Treg) are involved in the suppression of inflammation in IBS, ulcerative colitis, and Crohn's disease through the inhibition of T effector cells. It has been shown that T cells express high levels of CD25 Tregs in the colon in IBS patients. Therefore, any alteration in the frequency of Tregs may lead to recruitment of immune effectors which, consequently, results in inflammation [83, 84]. 

Intestinal epithelial cells recognize pathogens by way of pattern recognition receptors including Toll-like receptors (TLRs) and nucleotide-oligomerization-domain-(NOD) like receptors. They induce innate immune responses by the transcription and translation of antimicrobial proteins and the induction of proinflammatory cytokines and chemokines through the NF-*κ*B pathway [30, 70]. Ten TLRs have been reported in man so far, which recognized various microbial pathogens, including viruses, bacteria, fungi, and protozoa. A recent study has reported upregulation of TLR 4 and TLR 5 and down-regulation of TLR 7 and TLR 8 in IBS patients [28]. Increased levels of TLR 4 and TLR 5 indicate that their cognate ligands, LPS and flagellin, are also increased in IBS patients. Since the ligand for TLR 7 and TLR 8 is single-stranded RNA, decreased level of TLR 7/8 suggested that viral infection may also play an important role in the development of IBS-like symptoms. Such infections have been reported in relation to the development of PI-IBS. These data further support that increased permeability is present in at least a subgroup of IBS patients [28, 85]. 

*β*-defensin 2 is an antimicrobial protein secreted by intestinal epithelial cells and induced by TLR 4. Increased levels of *β*-defensin 2 have been reported in the intestine of patients with either IBS or ulcerative colitis [84, 85]. Several studies have shown that commensal bacteria may reduce inflammation, in part, by directly acting on dendritic cells to stimulate the induction of IL-10 and regulatory T cells (Treg). With an increase in the number of commensal bacteria, dendritic cells provide signals to lymph nodes to stimulate adaptive immune responses leading to induction of IgA antibodies that wrap the luminal antigens and, thus, prevent them from breaching the intestinal barrier and the inhibition of the systemic immune response. Dendritic cells can directly sample luminal pathogens without disruption of tight junctions [30, 55, 86]. Degranulation of mast cells releases histamine and other potent mediators that can influence the function of the enteric nervous system and smooth muscles, causing IBS-like symptoms [30]. In addition, a study has shown that mast cells are significantly increased in the caecum in patients with IBS [87]. Tryptase, a protease released by mast cells, has been reported to be significantly increased in the colonic mucosa of patients with IBS. Increased concentrations of serine protease have been reported in the stool of IBS subjects [30, 84].

### 5.5. Targeting the Microbiota

#### 5.5.1. Probiotics

The observation of dysbiosis in the gut microbiota, altered mucosal barrier function, activated immune responses, and SIBO support a role for bacteria in the pathogenesis of IBS [88]. Probiotics are live or attenuated microorganisms which, when administered in sufficient quantities, have been shown to improve gut epithelial integrity, as well as alleviate the symptoms of IBS [30, 88–90]. Previous studies have shown that the administration of adequate amounts of probiotics (live microorganisms) may alleviate the symptoms of IBS, suppress proinflammatory cytokines, and promote the integrity of the intestinal barrier [3, 31]. One study showed that the consumption of *Bifidobacterium infantis* 35624 was associated with proliferation of T regulatory cells, reduction of proinflammatory cytokines, down regulation of T cells, reduced expression of co-stimulatory molecules, and attenuation of NF-*κ*B [86]. In vitro, increased levels of proinflammatory cytokines (IL-12) and decreased concentrations of the anti-inflammatory cytokine (IL-10) by PBMCs have been reported in IBS patients. The ratio of IL-10/IL-12 was altered in IBS patients compared to healthy volunteers and the administration of *Bifidobacterium infantis* normalised this ratio [90]. Probiotics also inhibit adhesion of enteric pathogens to the wall of the gastrointestinal tract [30].

Probiotics should have the following characteristics: (1) they must survive in the gastrointestinal tract following passage and eventually reside in the colon, (2) they must not have a major adverse effect on other beneficial bacteria in the gut, (3) they should be hostile to mutagenic or pathogenic organisms in the gut, and (4) they must be stable genetically [91]. In clinical studies, probiotics have been shown to improve infectious or secretory diarrhea, traveller's diarrhea, and antibiotic-induced diarrhea via a number of mechanisms that may include direct effects on gastrointestinal motility and the enteric nervous system [30]. In a separate study, patients with IBS were treated with *Bifidobacterium infantis* or *Lactobacillus salivarius* (1E10) in malted milk or malted milk alone (as a placebo) for 8 weeks; there was a significant reduction in abdominal pain, discomfort, bloating, distension, and bowel movement difficulty in patients who received *Bifidobacterium infantis *compared with those who had placebo [90].

The use of multispecies probiotics has shown favorable effects in improving symptoms of IBS. VSL#3 contains a mixture of different bacterial species including *Lactobacillus* species (*L. casei, L. plantarum, L. acidophilus, and L. delbrueckii*), *Bifidobacterium species (B. longum, B. breve, and B. infantis*), and *Streptococcus thermophilus*. A randomized controlled trial showed that the oral administration of VSL#3, twice daily for 8 weeks, significantly reduced abdominal bloating, but not other parameters (colonic transit time, bowel dysfunction, abdominal pain, flatulence, or urgency) in a subgroup of diarrhea predominant IBS patients, when compared with placebo [92]. In a second study targeting 48 IBS patients with bloating, VSL#3 significantly reduced flatulence and colonic transit compared with the placebo group [93].

In summary, many clinical trials have investigated the therapeutic benefits of probiotics in patients with IBS. However, differences in duration of therapy, heterogeneity in species or strains of selected bacteria, and differences in characteristics of the enrolled patients have resulted in inconsistent results.

#### 5.5.2. Prebiotics

Prebiotics are nondigestible dietary supplements that affect the host by stimulating the growth of beneficial bacteria in the colon. Prebiotics have the capability to stimulate only microbes which are already residing in the gut [94]. Prebiotics are fermented by host bacteria and have been associated with a reduction in the level of triglyceride, improvement of the postprandial glucose level and a reduction in intestinal permeability [3, 95]. The fermentation of prebiotics leads to the production of SCFAs such as butyric acids, which can serve as energy source for intestinal epithelial cells [94]. When galactooligosaccharides are used as prebiotics, they are known to stimulate gut bifidobacteria in IBS patients and, thereby, reduce the symptoms of IBS [96]. A potential limitation of prebiotic treatment is that prebiotics undergo fermentation and could produce bloating and flatulence [97].

#### 5.5.3. Synbiotics

Synbiotics are defined as a combination of probiotics and prebiotics [98]. One study has shown that a combination of *Bifidobacterium spp.* and a prebiotic, inulin, significantly increased the quantity of *Bifidobacteria*. Furthermore, prebiotics also help passage of probiotics through the upper gastrointestinal tract and facilitate their establishment in the colon [91]. However, data on synbiotics in various gastrointestinal diseases including IBS is scanty.

#### 5.5.4. Prokinetics

Prokinetic drugs increase gastrointestinal motility. As impaired gut motility is associated with dysbiosis and SIBO, prokinetics could benefit IBS patients through an effect on the microbiota [99]. However, studies reporting the use of erythromycin for the treatment of IBS have shown limited efficacy [100]. Furthermore, domperidone and cisapride were not always effective for the treatment of IBS. In any event, cisapride has been withdrawn from the market due to adverse cardiac effect [101].

#### 5.5.5. Antibiotics

As discussed in previous sections, accumulating data support the role of bacteria in the etiology of IBS [102–104], and studies using antibiotics to target the intestinal microbiota to treat IBS are now emerging. In a double blind, randomized placebo-controlled study, neomycin was more effective than placebo in reducing IBS symptoms. However, the use of neomycin in the treatment of IBS has been limited by a marginal degree of efficacy above placebo and side effects [61].

Rifaximin, derived from rifamycin, is highly concentrated in the gut lumen and has little systemic absorption. It has been used in the treatment of traveller's diarrhea and SIBO. In a recent, large, double-blind, placebo-controlled trial, in which subjects were administered 550 mg rifaximin 3 times daily for 2 weeks and followed up for 10 weeks, there was a significant reduction in global IBS symptoms in the rifaximin group in comparison to placebo (40.8% versus 31.2%). In addition, there was a significantly greater reduction in bloating in those who received rifaximin compared to placebo (40.2% versus 30.3%) [89, 103, 105].

A combination of probiotics and antibiotics may play a beneficial role in the treatment of IBS symptoms [3]. Probiotics may increase the efficiency of antibiotics and reduce gastrointestinal pathogens by the production of antibacterial molecules including bacteriocins [3].

## 6. Summary and Conclusions

The literature on PI-IBS, SIBO, the relationship between gut microbiota and GI sensorimotor functions, and the potential for probiotics and antibiotics to alter these functions and to improve some of the symptoms of IBS, taken together, provide strong evidence in support of a major role for the gut microbiota in the pathogenesis of IBS. This concept represents a potential paradigm shift in our understanding of the underlying mechanism (for at least a subset of patients with IBS) from that of IBS as an entirely psychosomatic disorder to that of a more organic disorder related to an altered gut microbiota and low-grade inflammation. This could, ultimately, lead to a potential change in the management of IBS to strategies that alter the gut microbiota and inflammation.

## Figures and Tables

**Table 1:** Summary of the studies on the alteration in gut microbiota in IBS subjects.

S. No.	IBS (*n*)	Healthy (*n*)	Diagnostic criteria	Method	Outcome	Reference
1	11	8	Rome II	PCR and DGGE	Diversity of total bacteria along with *Lacto bacillus *was higher in IBS patients than healthy control	Ponnusamy et al. [1]
2	24	23	Rome II	Nucleic acid fractionation according to GC content, cloning followed by sequencing and qPCR	*Collinsella aerofaciens, Clostridium cocleatum, and Coprococcus eutactus *confirmed significant difference from that of healthy subjects	Kassinen et al. [18]
3	10	10	Rome III	Culture and q PCR	Significantly decreased quantity of aerobic bacteria in IBS than healthy control and increased concentration of *Lactobacillus* species in D-IBS patients than healthy control	Carroll et al. [19]
4	37	20	Rome II	DGGE and q PCR	Significant increase in the amount of *Pseudomonas aeruginosa* in IBS patients	Kerckhoffs et al. [33]
5	16	16	Rome II	RT-PCR-DGGE, Transcript analysis with the aid of affinity capture (TRAC)	Significantly decreased amount *Clostridium coccoides-E. rectale* in IBS-C	Maukonen et al. [106]
6	41	26	Rome II	FISH Analysis	Significantly decreased quantity of *Bifidobacterium catenulatum* in faecal and duodenal samples of IBS patients than healthy control	Kerckhoffs et al. [107]
7	44	34	Rome II	Culture and PCR	Significantly increased quantity of Enteraggroegative* Escherichia coli *in IBS-D	Sobieszczańska et al. [108]
8	20	15	Rome II	q PCR	Decreased quantity of *Clostridium thermosuccinogens *in IBS-D patients and increased quantity of *Ruminococcus torque *in IBS-D patients than healthy control. *Ruminococcus bromii* was more abundant in IBS-C than healthy control	Lyra et al. [109]
9	12	22	Rome II	% G + C profiling and fractioned DNA sequencing followed by q PCR	Significantly increased quantity of *Proteobacteria* and *Firmicutes* and reduced quantity of *Actinobacteria* and *Bacteroidetes* in IBS-D patients than healthy control	Krogius-Kurikka et al. [110]
10	25	25	Rome II	Culture	Significantly reduced number of Bifidobacterium and increased number of Enterobacteriaceae in IBS patients than healthy control	Si et al. [111]
11	47	33	Rome II	DGGE of 16S rRNA	Significant difference in gut microbiota in IBS patients and healthy control along with more variation in the gut microbiota in control than IBS subjects	Codling et al. [112]
12	26	26	Rome II	Culture and q PCR	Significantly increased quantity of *Lactobacillus* and *Veillonella* in IBS patients than control	Tana et al. [113]
13	10	10	Rome III	PCR and Pyrosequencing	Significantly increased number of *Bacteroidetes* and *Synergistetes* and reduced number of *Actinobacteria, Bacilli, Flavobacteria,* and *Epsilonproteobacteria* in IBS than control	Ng et al. [114]
14	22	22	Rome III	Metagenomics of 16S rRNA gene followed by PhyloChip hybridization and Pyrosequencing	Significantly greater abundance of class *γ*-Proteobacteria in IBS children than healthy control and *Haemophilus parainfluenzae* was prominent	Saulnier et al. [35]
15	62	46	Rome II	q PCR and microarray	Significantly 2-fold increased ratio of Firmicutes to Bacteroidetes in IBS patients comparison with the healthy control	Rajilić–Stojanović et al. [34]

IBS: irritable bowel syndrome; IBS-D: diarrhea predominant IBS; IBS-C: constipation predominant IBS; PCR: polymerase chain reaction; DGGE: denaturation gradient gel electrophoresis; q PCR: real time PCR; FISH: fluorescent in situ hybridization; TRAC: transcript analysis with the aid of affinity capture.

**Table 2:** Summary of prevalence of SIBO in IBS patients by different diagnostic methods.

Diagnostic method	N. of IBS patients	N. of controls	Percentage of SIBO in IBS subjects	Percentage of SIBO in controls	Reference
LHBT	76	40	44.7%	40.0%	Park et al. [115]
LHBT	43	56	65%	7%	Scarpellini et al. [116]
LHBT	127	—	43%	—	Carrara et al. [5]
LHBT	258	—	34.5%	—	Mann and Limoges-Gonzales [117]
LHBT	98	—	65%	—	Nucera et al. Lombardolll [118]
GHBT	59	37	23.7%	2.7%	Sachdeva et al. [119]
GHBT	98	—	36%	—	Reddymasu et al. [120]
GHBT	200	50	24.5%	6%	Lombardo et al.
GHBT	1921	—	31%	—	Ford et al. [121]
GHBT	130	70	16.1%	4.2%	Parodi et al. [122]
GHBT	225	100	11.1%	1%	Rana et al. [123]
GHBT	204	—	46%	—	Majewski et al. [124]
GHBT	96	—	45.8%	—	Cuoco and Salvangnini [125]
GHBT	65	102	31%	4%	Lupascu et al. [126]
GHBT	129	51	8.5%	2%	Ghoshal et al. [58]
Hydrogen	158	34	32.9%	17.9%	Grover et al. [127]
Breath test and culture of small bowel aspirate	162	26	4%	4%	Posserud et al. [56]

Abbreviations used: SIBO: small intestinal bacterial overgrowth; IBS: irritable bowel syndrome; LHBT: lactulose hydrogen breath test; GHBT: glucose hydrogen breath test.
